# Artificial Intelligence in the Intensive Care Unit

**DOI:** 10.1186/s13054-020-2785-y

**Published:** 2020-03-24

**Authors:** Guillermo Gutierrez

**Affiliations:** Pulmonary, Critical Care and Sleep Medicine Division, The George Washington University, Washington, DC, USA

## Abstract

This article is one of ten reviews selected from the Annual Update in Intensive Care and Emergency Medicine 2020. Other selected articles can be found online at https://www.biomedcentral.com/collections/annualupdate2020. Further information about the Annual Update in Intensive Care and Emergency Medicine is available from http://www.springer.com/series/8901.

## Introduction

**Fig. 1:**
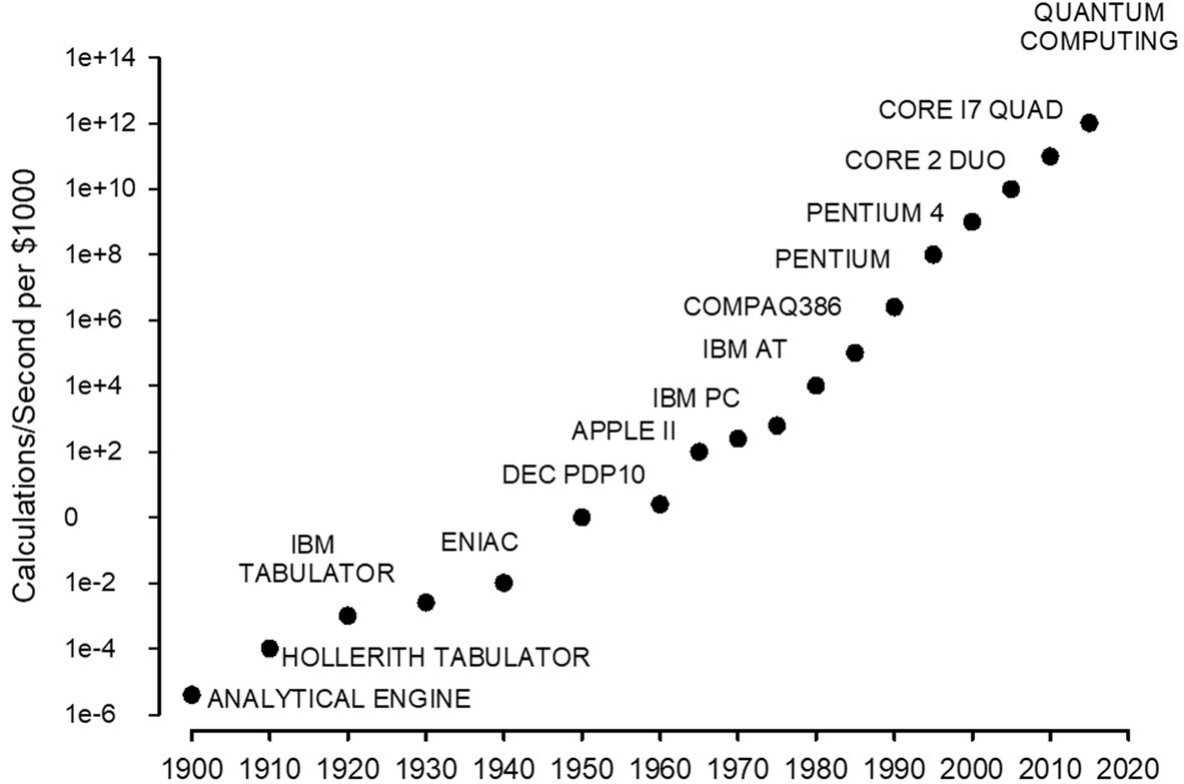
The growth of computer power, based on calculations per second purchased by $1000 USD (constant 2015) during the past century. Also shown are significant developments in technology associated with increases in computer power. Modified from https://www.flickr.com/photos/ jurvetson/25046013104 (with license). Original graph in Ray Kurzweil. “The singularity is near: When humans transcend biology,” p67, The Viking Press, 2006

The past century has witnessed a massive increase in our ability to perform complex calculations. The development of the transistor in the 1950s, followed by the silicone integrated circuit, accelerated those capabilities and gave rise to what is commonly known as Moore’s Law. According to this principle, the number of transistors packed into a dense integrated circuit doubles every 2 years. The corollary is that computation speed also doubles at 2-year intervals. Figure 1 is a graphical interpretation of Moore’s Law, showing an exponential increase in computational power, in terms of calculations per second that can be purchased with $1000 (constant US, 2015). According to that graph, computing power has increased by a factor of 10^18^ from the mechanical analytical engine of the early 1900s to today’s core I7 Quad chip found in personal laptop computers.

The growth in computing power was made possible by the relentless downsizing of integrated circuits, with some components being produced in the sub-100 nm range. As we approach the physical limits of silicone chip downsizing, other materials are being developed. A likely candidate is the carbon nanotube, composed of a single sheet of carbon atoms arranged in a hexagonal pattern. When rolled into itself, the sheet becomes a tube approximately 2 nm in diameter, capable of forming different circuit elements. This nascent technology, along with the development of quantum computing, assures the durability of Moore’s Law well into the future.

As processors grew in power, and personal computers became ubiquitous appliances, the stage was set for the development of the Internet, a digital network that morphed from the ARPANET, a communication structure designed by the U.S. Advanced Research Projects Agency (ARPA) to transfer information among computers located at remote distances. The internet promoted the free dissemination of software and provided the impetus for computer scientists to develop powerful algorithms aimed at simulating human intelligence.

According to the Encyclopedia Britannica, artificial intelligence (AI) refers to a system “endowed with the intellectual processes characteristic of humans, such as the ability to reason, discover meaning, generalize, or learn from past experience.” AI computer systems are able to perform tasks normally requiring human intelligence and that are considered “smart” by humans. AI systems act on information, such as controlling a self-driving automobile or influencing consumer shopping decisions.

In the area of medicine, AI has been used in drug discovery, personalized diagnostics and therapeutics, molecular biology, bioinformatics, and medical imaging. AI applications are also capable of discerning patterns of disease by scrutinizing and analyzing massive amounts of digital information stored in electronic medical records. In a recent proposal aimed at regulating AI software in medical devices, the U.S. Food and Drug Administration states that “Artificial intelligence-based technologies have the potential to transform healthcare by deriving new and important insights from the vast amount of data generated during the delivery of healthcare every day” [1].

### Machine Learning

Human intelligence is defined by the mental capability to think abstractly, use reason to solve problems, make plans, comprehend complex ideas, and learn from experience [2]. Much of human intelligence involves pattern recognition, a process that matches a visual or other type of stimuli, to similar information stored in our brains. Although endowed with abstract thinking and capable of sublime leaps in imagination, humans have a limited capacity for memory. It is estimated that the brain cannot store more than four “chunks” of short-term memory at any one time [3]. Moreover, humans find it difficult to think in terms of *n*-dimensional spaces or visualize patterns embedded into large quantities of data. Conversely, computers have vast memory storage, excel at handling multidimensional problems and can discern even small or “fuzzy” associations within massive data collections.

**Fig. 2:**
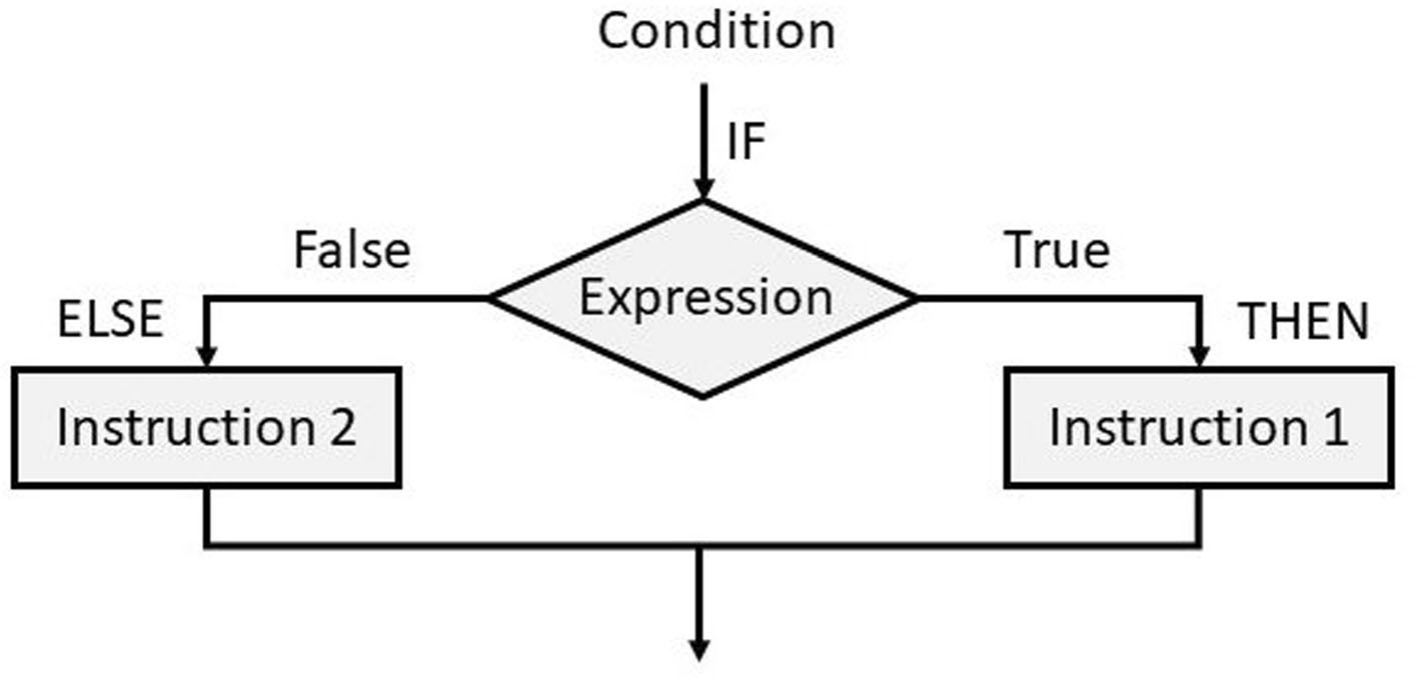
A logical IF expression. The condition is evaluated by the expression, and Instruction 1 is executed if TRUE, otherwise, Instruction 2

The use of computers to guide the treatment of critically ill patients is not a new concept. With uneven results, computerized systems have been proposed in the past to monitor ICU patients [4], manage patients on mechanical ventilators [5, 6], guide care in patients with acute respiratory distress syndrome (ARDS) [7], and manage arterial oxygenation [8]. These early computer systems were programmed with highly specific and sequential IF/THEN/ELSE logical expressions that assessed the validity of a condition based on accepted physiological principles and/or clinical experience (Fig. 2). According to these expressions, IF a given condition was judged to be “true,” THEN the program executed instruction 1, ELSE, it executed instruction 2.

AI is based on a fundamentally different approach to traditional computer programming. Instead of instructing the computer to evaluate a given condition, or to perform a specific task according to detailed programmed instructions, AI algorithms, in a manner similar to the way children absorb knowledge, learn from exposure to numerous examples. AI algorithms establish their own rules of behavior and can even improve on their “intelligence” by incorporating additional experiences resulting from the application of these rules.

**Fig. 3:**
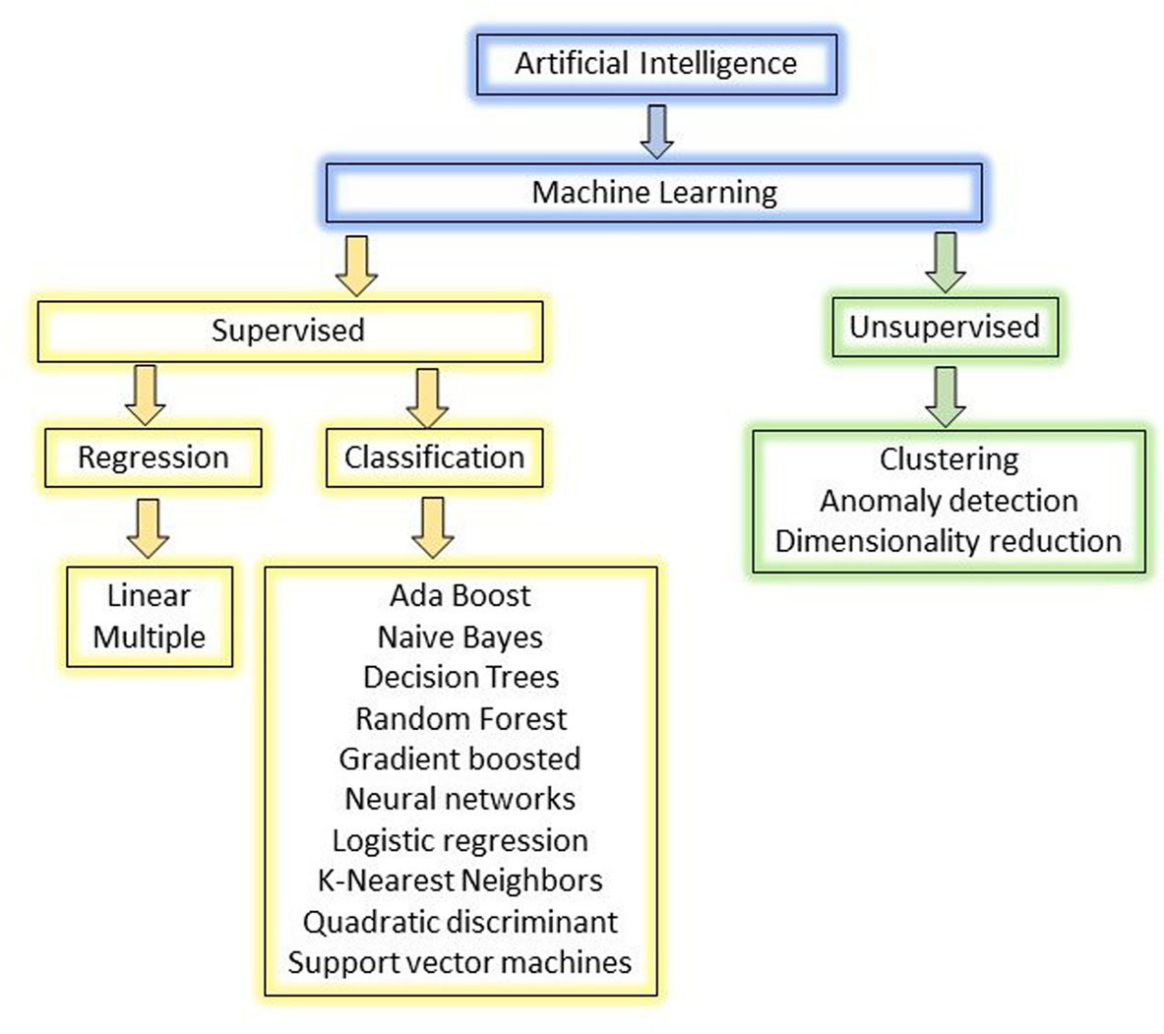
Machine learning is a branch of artificial intelligence encompassing two major approaches: supervised and unsupervised learning. Shown under each branch are algorithm types used in model development

Machine learning is a subset of AI in which machines learn or extract knowledge from the available data, but do not act on the information. Machine learning combines statistical analysis techniques with computer science to produce algorithms capable of “statistical learning.” Broadly speaking, there are two types of machine learning structures: supervised and unsupervised (Fig. 3).

### Supervised Machine Learning

The objective of supervised machine learning is to develop an algorithm capable of predicting a unique output when provided with a specific input. In other words, the machine is shown examples of input (*x*) and its corresponding output (*y*), such that *y* = *f*(*x*). Machine learning is predicated on large sets of data containing myriad examples that relate one or several input variables to a single output. The expectations are that the resulting algorithm will deliver accurate predictions when exposed to new and never before seen data. Supervised learning requires a great deal of human effort when building large datasets to train and test the algorithm. There are two major types of supervised learning: regression and classification.

### Regression Learning

Most clinicians are familiar with regression analysis, a statistical technique producing a mathematical expression relating one input variable to another (linear regression) or many input variables to one dependent variable (multiple regression). In regression analysis, the output is a continuous function of the input. In other words, the predicted variable will change in concert with the input variables. Regression is used commonly to test hypotheses involving causal relationships, with the choice of model being based on its significance and goodness of fit.

### Classification Learning

Classification supervised learning is a form of pattern recognition designed to predict a single, nonnumerical output, or “class,” from a predefined list of possibilities. Classifier algorithms are trained with many lines of data, with each line having several input variables and one desired output. For example, a model designed to identify a breed of dog may be trained with data listing their traits or characteristics, e.g., height, type of hair, and length of tail. Each line will be associated with a specific breed. Once trained, the model can be asked to predict the dog breed when given new set of input variables. Two important steps are needed to build a classifier model. The first is to establish the number of classes the model will be required to identify. The second is to identify the number of variables required to describe the classes. Fewer variables and classes require less training data and result in simpler and more accurate models. The simplest classification model is the binary kind, in which the model is asked to choose between a “Yes” and a “No” answer.

Classes may consist of physical objects (chair, table, etc.), medical conditions (e.g., sepsis, ARDS, chronic obstructive pulmonary disease [COPD], etc.), clinical or physiological observations (e.g., different types of arrhythmia or ventilator asynchronies). Each class is associated with a number of input variables common to all classes. In machine learning parlance, input variables are known as “features,” with each line of data, or “instance,” containing several features and a single class.

**Fig. 4:**
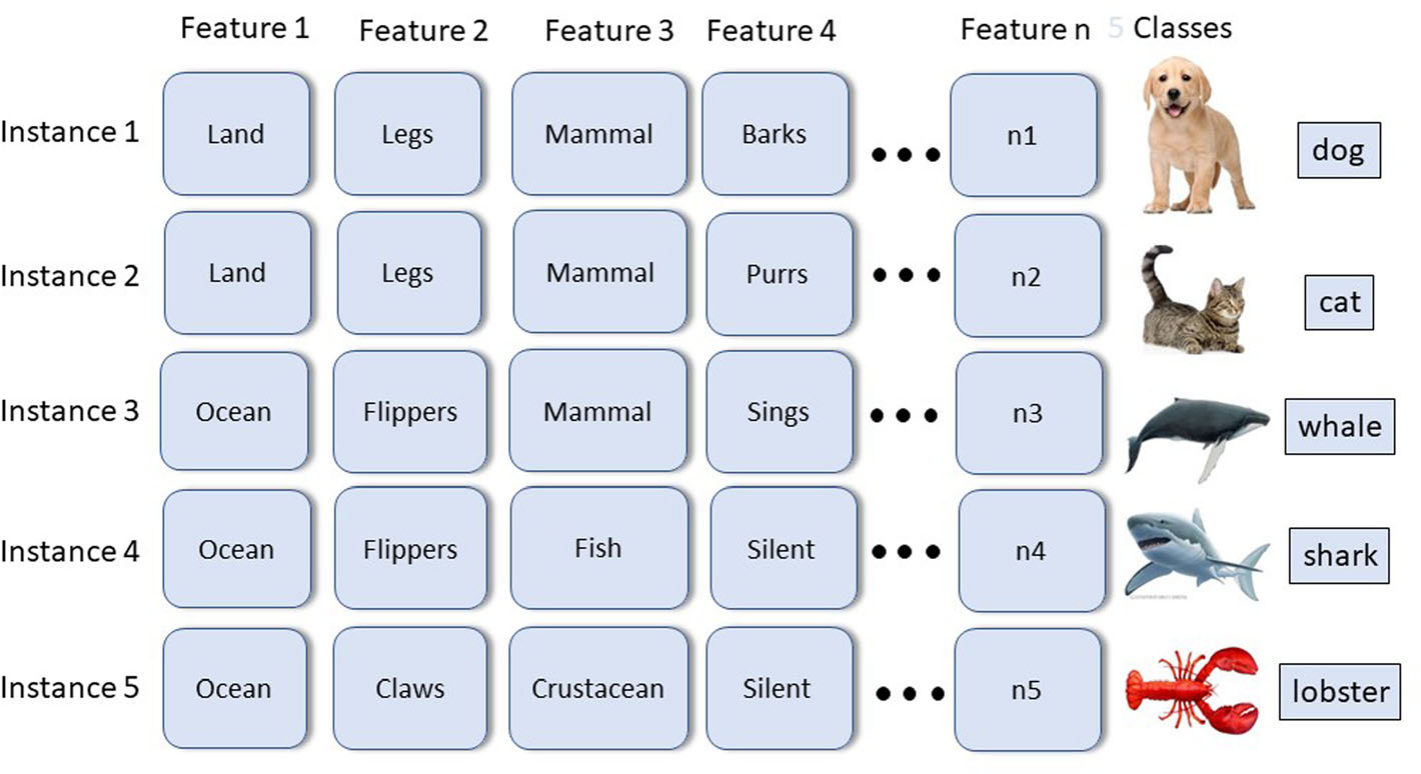
An example of a classification problem showing features describing five classes of animal. Each line represents an instance

Let us say we want to develop a classifier algorithm to identify five different kinds of animal (Fig. 4). In this example, each line of data has one animal class and several features to describe the animal’s characteristic, such as sea or land dwelling, fish or mammal. This is a very simple example having only one instance per class. The model, therefore, would be totally inadequate if its purpose were to differentiate among different dog or cat breeds. In that case, many more instances would be needed to describe different types of dogs and cats. The more specific one wishes to be, the more features are needed to describe the classes. On the other hand, increasing the number of features results in complex models that require greater computing power and longer time to run, a condition termed “the curse of dimensionality.” An important guiding principle in machine learning is the truism that “less is best.”

In mathematical terms, a feature matrix contains *n* features and *m* instances, and it is associated with an *m* length classification vector:

$$ {\displaystyle \begin{array}{l}x11x12\dots xn1=y1\\ {}x12x22\dots x2n=y2\\ {} xm1x2m\dots xn m= ym.\end{array}} $$

#### Developing a classifier model

**Fig. 5:**
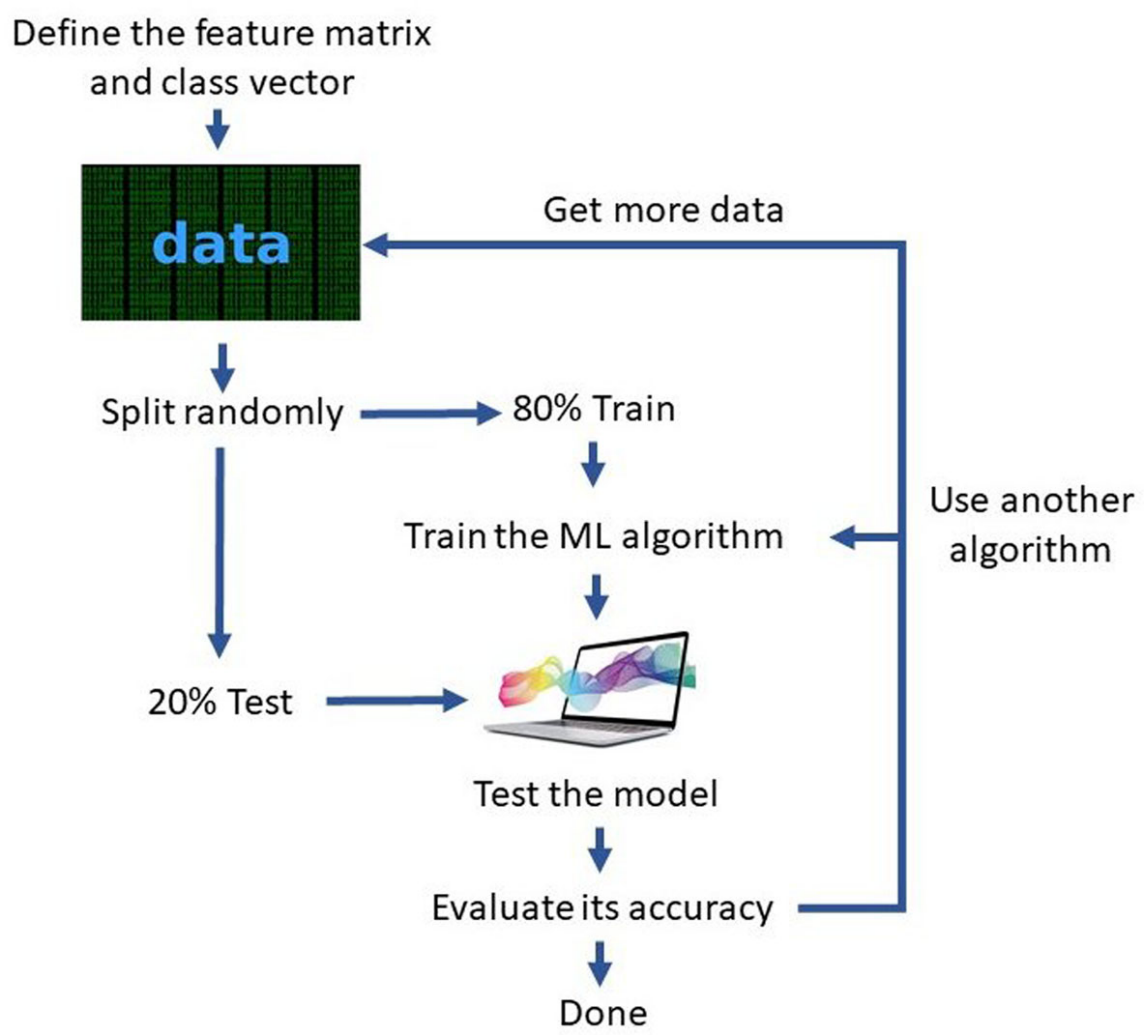
The process of creating a machine learning (ML) model

Perhaps the most important step in developing a machine learning model is to have a clear definition of the problem and to determine its suitability for machine learning. The next step is to determine the size of the feature matrix and the classification vector (Fig. 5). Whereas humans develop generalized concepts on the basis of just a few examples, training a machine learning algorithm requires large quantities of data. The creation of a large feature matrix with its classification vector is accomplished by gathering as many instances as possible. Once satisfied that we have collected an adequate number of examples to be presented to the computer, we split the feature matrix into a “training” dataset, for model development, and a “test” dataset. The data are split by a random process that assigns instances from the original data to each dataset. A common practice is to use 70% or 80% of the data for training and the remainder for testing.

The purpose of the “test” dataset is to assess the algorithm’s accuracy when exposed to never before seen data. Accuracy is defined as the percentage of correct answers made by the algorithm on the unknown “test” dataset. Should accuracy fall below a chosen expected value, we can choose to gather more “training” data or to use another type of machine learning algorithm altogether.

Several types of classifier algorithms may be used to create the machine learning model. Among them are decision trees, random forests, k-nearest neighbors, and many others (Fig. 3). A popular type of classifier algorithm is the neural network, modeled on the way human neurons are thought to process information. The basic element of the neural network, the perceptron, produces a single binary output from several inputs. A neural network results from the interacting of several perceptrons. Advanced machine learning systems encompassing several layers of stacked complex neural networks are called deep learning.

It is beyond the purpose of this chapter to describe the theory and application of these algorithms (listed in Fig. 3), but the reader interested in pursuing this line of investigation can access “scikit-learn” (https://scikit-learn.org/stable/), an open source machine learning library written with the Python programming language (https://www.python.org/). This library of programs makes it relatively easy to develop classification supervised machine learning algorithms.

When building a classifier model, it is imperative to generalize its utility to make accurate predictions using both the “training” and the “test” datasets. One should beware of models of high complexity that may conform closely to the “training” set, but have poor accuracy when applied to the “test” dataset, a phenomenon called “overfitting.”

### Unsupervised Machine Learning

In this type of machine learning, no instructions are given to the algorithm on how to process the data. Instead, the computer is asked to extract knowledge from a large set of unclassified data with no known output or a set of rules. Given the lack of label information, a major challenge for the investigator when evaluating an unsupervised algorithm is how to determine the utility of the results, or whether the right output has been achieved. Unsupervised algorithms, however, can be very useful in exploratory attempts to understand large collections of data. The techniques most commonly used are clustering, anomaly detection, and dimensionality reduction.

In clustering, algorithms are asked to identify or partition large data sets into subsections and patterns sharing similar characteristics. In anomaly detection the algorithm is asked to detect atypical patterns in the dataset, such as searching for outliers. Dimensionality reduction is useful when analyzing data having many features, or dimensions. These algorithms may be able to present the data in a simpler form, summarizing its essential characteristics and making it easier for humans or other machine learning algorithms to understand.

An important point to keep in mind is that no machine learning algorithm, regardless of its accuracy, is the only possible choice for a model. Other algorithms may be capable of providing a good fit and derive additional useful inferences from the data. For those wishing to delve deeper into the development of machine learning models, a good source of information is the book by Müller and Guido [9] and the website (https://www.geeksforgeeks.org/learning-model-building-scikit-learn-pythonmachine-learning-library/).

### AI Applications in Critical Care

There are numerous opportunities in the hospital setting to apply AI. Unsupervised machine learning techniques have been used to explore massive amounts of data encoded in electronic medical records. Models have been developed to obtain important information in a patient’s chart [10] and identify high-cost patients [11]. Supervised machine learning algorithms, given their potential for automated pattern recognition of images, have proven their utility in radiology [12] and histopathology [13]. Machine learning has been used extensively in the fields of surgery, as it pertains to robotics [14], in cardiology [15] for early detection of heart failure [16], and in cancer research to classify tumor types and growth rates [17].

Although the introduction of machine learning to the ICU is in its infancy, several studies have already been published describing the application of this technology in the management of the critically ill patient. Some have used large population datasets to predict length of stay, ICU readmission and mortality rates, and the risks of developing medical complications or conditions such as sepsis and ARDS. Other studies have dealt with smaller datasets of clinical and physiological data to aid in the monitoring of patients undergoing ventilatory support.

### Length of Stay

Houthooft et al. [18] trained a support vector machine model to forecast patient survival and length of stay using data from 14,480 patients. The model’s area under the curve (AUC) for predicting a prolonged length of stay was 0.82. This is in contrast to a clinical study showing the accuracy of physicians to be only 53% when predicting ICU length of stay [19]. A hidden Markov model framework applied to physiological measurements taken during the first 48 h of ICU admission also predicted ICU length of stay with reasonable accuracy [20]. The problem of ICU readmission was investigated with a neural network algorithm applied to the Medical Information Mart for Intensive Care III (MIMIC-III) database. This is an open source, freely available database collected from patients treated in the critical care units of the Beth Israel Deaconess Medical Center between 2001 and 2012. The algorithm was able to identify patients at risk of ICU readmission with 0.74 sensitivity and AUC of 0.79 [21].

### ICU Mortality

Awad et al. [22] applied several machine learning algorithms, including decision trees, random forest, and naïve Bayes to 11,722 first admission MIMIC-II data to predict ICU mortality. Features included demographic, physiological, and laboratory data. These models outperformed standard scoring systems, such as APACHE-II, sequential organ failure assessment (SOFA), and Simplified Acute Physiology Score (SAPS), a finding that was confirmed by the same group in a follow-up study using time-series analysis [23]. A Swedish system using artificial neural networks applied to >200,000 first-time ICU admissions also showed superior performance in predicting the risk of dying when compared to SAPS-3 [24]. Machine learning models have also been proposed to predict mortality in trauma [25] and pediatric ICU patients [26].

The abovementioned ICU survival models, while offering improved performance when compared to standard mortality prediction scoring systems, are somewhat cumbersome to use, require a large number of variables and have yet to be tested prospectively.

### Complications and Risk Stratification

Yoon et al. [27] developed a method to predict instability in the ICU based on logistic regression and random forest models of electrocardiogram (EKG) measures of tachycardia, reporting an accuracy of 0.81 and AUC of 0.87. The publication of the study is accompanied by an excellent and highly recommended editorial by Vistisen et al. [28] that thoroughly analyzes the strengths and pitfalls of machine learning methods as predictors of complications in the ICU.

A recent study applied a random forest classifier to over 200,000 electronic health records of hospitalized patients to predict the occurrence of sepsis and septic shock. Although the algorithm was highly specific (98%), it only had a sensitivity of 26%, severely limiting its utility [29]. Other studies have been published describing the use of machine learning models in generating patient-specific risk scores for pulmonary emboli [30], risk stratification of ARDS [31], prediction of acute kidney injury in severely burned patients [32] and in general ICU populations [33], prediction of volume responsiveness after fluid administration [34] and identification of patients likely to develop complicated *Clostridium difficile* infection [35].

### Mechanical Ventilation

Whereas present day mechanical ventilators work exceedingly well in delivering air to diseased lungs, they are “feed-forward” or open loop systems where the input signal, or mode of ventilation, is largely unaffected by its output, the adequacy of ventilation. As such, ventilators lack the capacity to assess the patient’s response to the delivered breath. A desirable solution is the development of the autonomous ventilator, a device that could monitor the patient’s response to ventilation continuously, while adjusting ventilatory parameters to provide the patient with a comfortable, optimally delivered breath. Although we are far from this ideal device, significant strides are being made toward making it into a reality.

Over the past decade, there has been considerable interest in detecting and classifying patient-ventilator asynchrony, a phenomenon indicating the degree of coupling or response of the patient to ventilatory support [36]. Machine learning methods of detecting patient-ventilator asynchrony have been based on morphological changes of the pressure and flow signals. Chen et al. [37] developed an algorithm to identify ineffective efforts from the maximum deflection of the expiratory portion of airway pressure and flow. Ineffective effort was present in 58% of the 24 patients enrolled in their study. Analysis of 5899 breaths yielded sensitivity and specificity for the detection of ineffective efforts >90%. An algorithm developed by Blanch at al [38]. compared a theoretical exponential expiratory flow curve to actual flow tracings. A deviation exceeding 42% was considered indicative of ineffective effort. They compared the predictions of the algorithm in a random selection of 1024 breaths obtained from 16 patients, to those made by five experts and reported 91.5% sensitivity and 91.7% specificity with 80.3% predictive value. As proof-of-concept, this group also reported monitoring airway signals in 51 mechanically ventilated patients and were able to predict the probability of an asynchrony occurring from one breath period to the next using a hidden Markov model [39]. The system used in these trials has been commercialized as Better Care®, and it is capable of acquiring, synchronizing, recording, and analyzing digital signals from bedside monitors and mechanical ventilators [38].

Rhem et al. [40] and Adams et al. [41] developed a set of algorithms to detect two types of asynchrony associated with dynamic hyperinflation, double triggering, and flow asynchrony. Based on a learning database of 5075 breaths from 16 patients, they developed logical operators to recognize double triggering based on bedside clinical rules. Dynamic hyperinflation was identified from the ratio of exhaled to inhaled tidal volume. The algorithms were validated with data drawn from another patient cohort (*n* = 17), resulting in sensitivity and specificity >90%.

Sottile at al [42]. evaluated several types of machine learning algorithms, including random forest, naïve Bayes, and AdaBoost on data recorded from 62 mechanically ventilated patients with or at risk of ARDS. They chose 116 features based on clinical insight and signal description and were able to determine the presence of synchronous breathing, as well as three types of patient-ventilator asynchrony, including double triggering, flow limited and ineffective triggering, with an AUC >0.89. The authors did acknowledge that their algorithm does not identify all types of patient-ventilator asynchrony, in particular premature ventilator terminated breaths, or cycling asynchronies.

Gholami et al. [43] trained a random forest classifier algorithm from a training data set produced by five experts who evaluated 1377 breath cycles from 11 mechanically ventilated patients to evaluate cycling asynchronies. Patients were ventilated with pressure-controlled volume ventilation. The model accurately detected the presence or absence of secondary synchrony with a sensitivity of 89%. Mulqueeny et al. [44] used a naïve Bayes machine learning algorithm with 21 features, including measures of respiratory rate, tidal volume, respiratory mechanics and expiratory flow morphology to a dataset of 5624 breaths manually classified by a single observer, resulting in an accuracy of 84%, but a sensitivity of only 59%. Loo et al. [45] trained a convolutional neural network with 5500 abnormal and 5500 normal breathing cycles aimed at developing an algorithm capable of separating normal from abnormal breathing cycles, reporting 96.9% sensitivity and 63.7% specificity.

### The Issue of Accuracy Versus Reliability

The accuracy of a machine learning algorithm is judged by its ability to correctly predict the unseen test dataset. Models are created and tested with instances culled from the same data population, and it is common to find reports of algorithms having very high accuracy scores in the machine learning literature. Given a judicious selection of features, a sufficiently large number of instances, and a wise choice of algorithm, the most likely outcome will be a highly accurate model. If the data are true and verifiable, the model’s predictions are also bound to be reliable. On the other hand, when a model trained with untested or faulty data is presented with data drawn from the same population, the predictions are likely to be accurate but totally unreliable. As some have succinctly put it, rubbish in, rubbish out.

This begs the question of what are the limits of model reliability. Whereas AI is able to consider numerous variables and minimize human bias in data classification, it cannot insure model reliability. Therefore, the greatest challenge when creating a clinical machine learning model lies in identifying the gold standard to be used in the classification. A great deal of what we see and do in medicine is highly subjective, and unanimity of opinion is seldom found among intensivists. For example, a study [46] on interobserver reliability of clinicians in diagnosing ARDS according to the Berlin definition found only a moderate degree of reliability (kappa = 0.50). The main driver of the variability was the interpretation of chest radiographs. Similar findings were noted in clinicians evaluating optic disk photographs for glaucoma (kappa 0.40–0.52) [47]. It is therefore unlikely that model reliability in the ICU will ever exceed 60–70%, even in the best of hands.

## Conclusion

Experienced intensivists excel at collecting, classifying, and analyzing snapshots of clinical information to expeditiously reach a diagnosis and decide on treatment options. In the data-intensive environment of today’s ICUs, however, intensivists must cope with a relentless flow of information, some of it useful, most of it not. According to a thoughtful essay by Alan Morris [48], intensivists must contend with no less than 236 variables when caring for patients on ventilatory support. The ability to catalog, correlate, and classify these variables on a continuous basis lies well beyond the capabilities of even the most knowledgeable and perceptive of clinicians.

The judicious application of AI technology can be of assistance in helping us deal with information overload. Machine learning algorithms have been used to analyze data stored in electronic medical records to predict ICU mortality and length of stay. They also have furthered our understanding of populations who may be at risk of disease progression or likely to experience medical complications. These retrospective studies, useful as they may be in the early identification and stratification of patients, represent only the low-lying fruit in AI research.

A more difficult task, but perhaps one with far greater potential, is the development of intelligent machine learning monitors capable of continuously assessing the human response to critical illness with a high degree of certainty. The development of such monitors will provide the knowledge and experience needed for the creation of the semi-autonomous ICU, an environment where intelligent machines provide most of the care delivered today by humans.

The full potential of AI will be realized once it becomes a trustworthy clinical adjunct to intensivists. By helping us cope with information overload, AI endowed machines may allow our faculties of reflection, imagination, and compassion to come to the fore when caring for fellow humans in distress. The future of AI in the ICU is indeed bright. As with all new technologies, there will be zealots and pharisees, ups and downs, elations and disappointments, as well as thorny ethical quandaries. I have no doubt, however, that AI is here to stay, and it behooves us to become familiar with this technology for the betterment of our patients.

## Data Availability

Not applicable.
